# Supplementary material for: Genes Involved in Immune Reinduction May Constitute Biomarkers of Response for Metastatic Melanoma Patients Treated with Targeted Therapy
Source: Biomedicines. 2022 Jan 26;10(2):284. doi: 10.3390/biomedicines10020284 (PMC8869294; doi:10.3390/biomedicines10020284)
Supplement: Supplementary file 1 [file biomedicines-10-00284-s001.zip › biomedicines-1515167-supplementary.pdf]

Appendix- Supplementary Material

1. Patient Flow Chart:

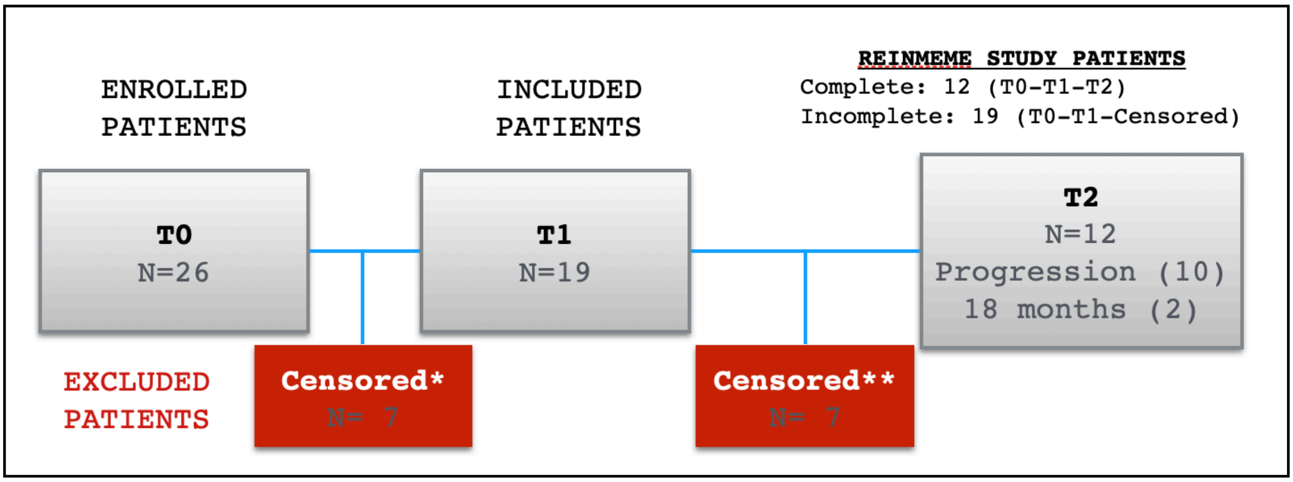

Figure S1. *REINMEME STUDY. Flow chart patients.*

*Censored patients: 2 pt with technical problems with samples, non-melanoma; 2 pt did not extract the blood sample, 1 pt with falsely diagnosed metastatic melanoma (sarcoid reaction). **Censored patients due to extreme clinical deterioration that conditioned their withdrawal of consent.

2. Survival analysis:

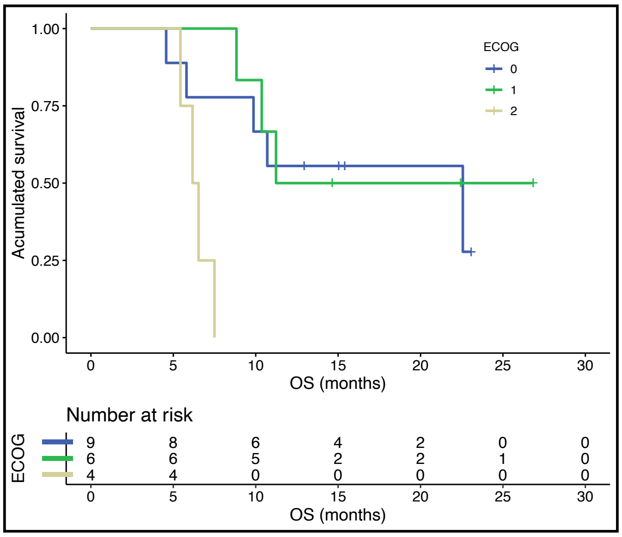

Figure S2. Kaplan-Meier Overall Survival Analysis by ECOG (*p<0.01*)

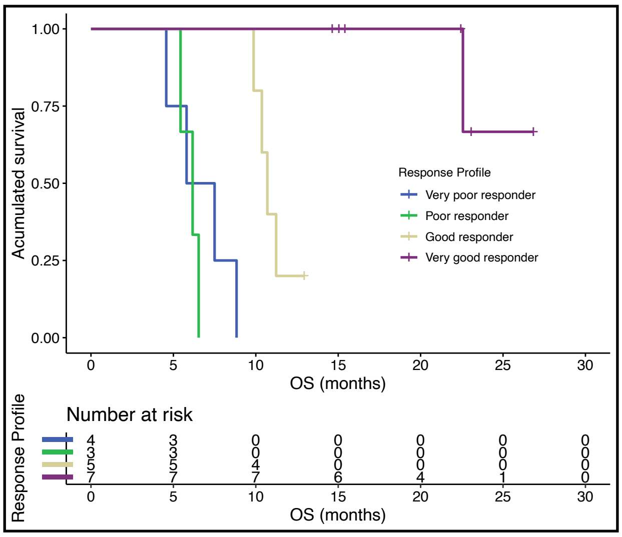

Figure S3. Kaplan-Meier Overall Survival Analysis by Response Profile (*p<0.001*)

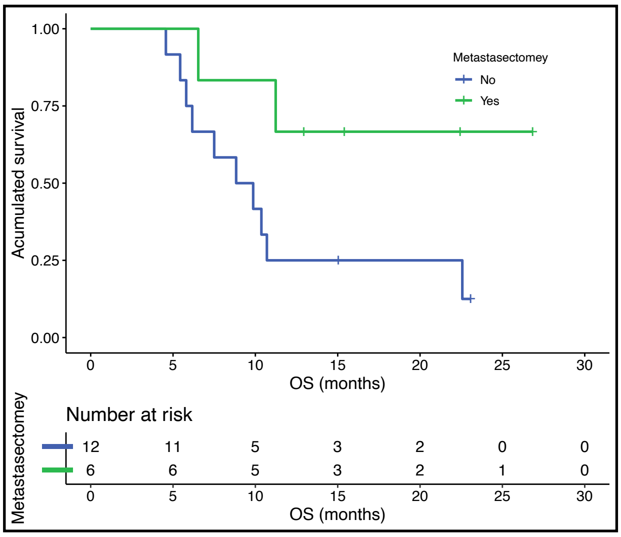

Figure S4. Kaplan-Meier Overall Survival Analysis by metastasectomy (*p=0.061*)

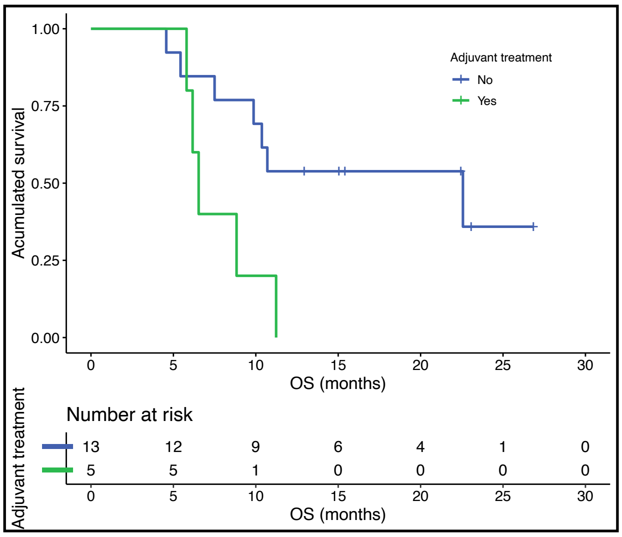

Figure S5. Kaplan-Meier Overall Survival Analysis by Adjuvant treatment (*p=0.024*)

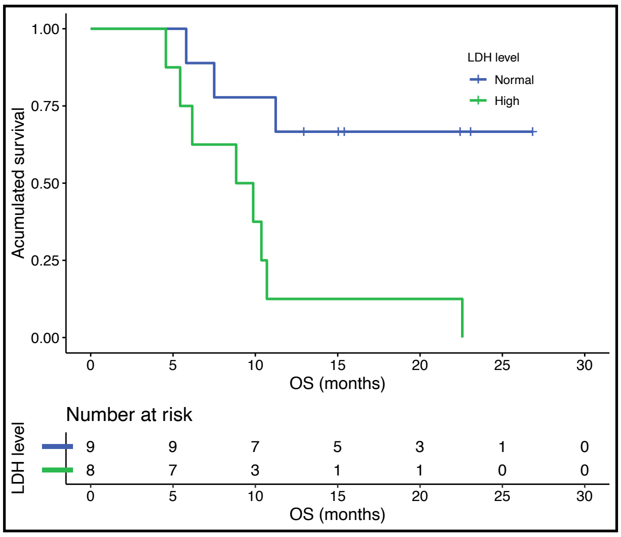

Figure S6. Kaplan-Meier Overall Survival Analysis by LDH (*p=0.008*)

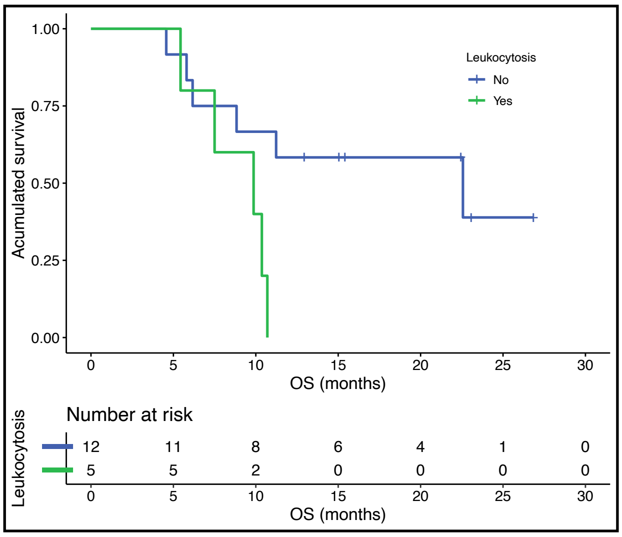

Figure S7. Kaplan-Meier Overall Survival Analysis by leukocytosis (*p=0.051*)

3. Tables

3.1. Table S1. Univariate Analysis (OS)

Patient features		p	Hazard Ratio (CI 95%)
Gender		0,461	1.58 (0.47 - 5.32)
ECOG		0, 004	9.99 (1.61 - 62.13)
Comorbidities<br>Allergies<br>Other medical conditions		 0,938<br>0,342	 0.94 (0.2 - 4.36)<br>Not Applicable
**Basal conditions**		**p**	**Hazard Ratio (CI 95%)**
Previous treatment<br>Primary tumor resection<br>Metastasectomy<br>Adyuvant treatment		 0,536<br>0,061<br>0,024	 0.68 (0.2 - 2.29)<br>0.26 (0.06 - 1.19)<br>3.78 (1.11 - 12.9)
Analitics<br>High LDH<br>High dNLR<br>Lymphocytes deplection<br>Leucocytosis		 0,008<br>0,334<br>0,861<br>0,051	 5.37 (1.37 - 21.13)<br>1.91 (0.5 - 7.23)<br>0.83 (0.1 - 6.61)<br>3.78 (0.99 - 14.4)
**Treatment and response**		**p**	**Hazard Ratio (CI 95%)**
Type of treatment (iBRAF/iMEK)		0,282	0.89 (0.26 - 3.07)
Dose reduction		0,181	0.46 (0.15 - 1.47)
Toxicity		0,255	0.19 (0.02 - 1.99)
Response to treatment		0,198	0.4 (0.1 - 1.62)
**Tumor features**		**p value**	**Hazard Ratio (CI 95%)**
Final TNM Stage		0,838-0,977	1.17 (0.14 - 9.97)
Ulceration		0,581	1.46 (0.38 - 5.67)
Primary tumor location		0,887	0.54 (0.09 - 3.29)
Metastatic location<br>Number<br>CNS<br>Bone		 0,140<br>0,874<br>0,197	 4.15 (0.53 - 32.22)<br>0.9 (0.24 - 3.34)<br>2.21 (0.64 - 7.63)
BRAF mutation		0,320	Not applicable

3.2. Table S2. Multivariate Analysis (OS)

Clinical variables		P value	Hazard Ratio (CI 95%)
ECOG		0,5997	1,828 (0,192 - 17,415)
TNM		0,0721	0,112 (0,010 - 1,217)
Metastasectomy		0,0516	0,018 (0,0003 - 1,029)

Number of metastatic		0,0649	39,45 (0,797-1952,9)
LDH		0,9823	0,976 (0,114-8,325)
High dNLR		0,5459	1,783 (0,273-11,658)
Toxicity		0,7481	1,396 (0,182 - 10,679)

	ECOG	Metastasectomy	Adjuvant	Leukocyte count	LDH stratification	Responder	OS1	PFS	OS
Green	−0.58 (0.01)	−0.12 (0.3)	−0.23 (0.2)	−0.65 (0.003)	−0.43 (0.06)	0.37 (0.9)	0.5 (1)	0.48 (1)	0.46 (1)
Blue	0.19 (0.7)	0.16 (0.7)	−0.029 (0.5)	0.22 (0.8)	0.098 (0.6)	−0.34 (0.1)	−0.21 (0.2)	−0.34 (0.1)	−0.22 (0.2)
Brown	0.41 (0.9)	0.049 (0.6)	0.062 (0.6)	0.32 (0.9)	0.3 (0.9)	−0.39 (0.08)	−0.28 (0.2)	−0.36 (0.1)	−0.24 (0.2)
Turquoise	0.48 (1)	0.057 (0.6)	0.49 (1)	0.12 (0.7)	0.11 (0.7)	−0.56 (0.01)	−0.35 (0.1)	−0.25 (0.2)	−0.29 (0.2)
Yellow	0.72 (1)	0.01 (0.5)	0.015 (0.5)	0.47 (1)	0.38 (0.9)	−0.27 (0.2)	−0.3 (0.1)	−0.26 (0.2)	−0.21 (0.2)
Grey	0.087 (0.6)	0.41 (0.9)	0.27 (0.8)	−0.25 (0.2)	−0.051 (0.4)	−0.1 (0.4)	−0.086 (0.4)	−0.13 (0.3)	−0.21 (0.2)

Table S3a. Module-trait relationships (T0). In each row, module of colors with different gene co-expression. In each column, clinical variables. The relationship between the two is shown and the statistical significance (Benjamini-Hochberg adjusted p-value) in brackets.

	ECOG	Metastasectomy	Adjuvant	Lukocyte count	LDH stratification	Responder	OS1	PFS	OS
Red	0.1 (0.6)	−0.11 (0.3)	0.17 (0.7)	−0.11 (0.3)	0.41 (0.9)	0.12 (0.7)	0.1 (0.6)	0.0026 (0.5)	0.16 (0.7)
Turquoise	−0.43 (0.05)	0.029 (0.5)	−0.19 (0.2)	−0.069 (0.4)	0.13 (0.7)	0.43 (1)	0.52 (1)	0.34 (0.9)	0.57 (1)
Black	0.011 (0.5)	0.43 (1)	−0.15 (0.3)	−0.084 (0.4)	−0.35 (0.1)	0.29 (0.9)	−0.09 (0.4)	0.2 (0.8)	−0.047 (0.4)
Brown	−0.4 (0.06)	0.14 (0.7)	−0.5 (0.02)	−0.038 (0.4)	−0.65 (0.003)	0.32 (0.9)	0.27 (0.8)	0.38 (0.9)	0.27 (0.8)
Blue	−0.12 (0.3)	−0.17 (0.3)	−0.068 (0.4)	0.3 (0.9)	−0.13 (0.3)	−0.26 (0.2)	−0.37 (0.08)	−0.3 (0.1)	−0.42 (0.05)
Green	0.2 (0.8)	−0.35 (0.09)	0.44 (1)	0.25 (0.8)	0.47 (1)	−0.49 (0.03)	−0.48 (0.03)	−0.58 (0.008)	−0.53 (0.02)
Grey	0.028 (0.5)	−0.28 (0.2)	−0.33 (0.1)	0.3 (0.9)	0.39 (0.9)	−0.15 (0.3)	−0.2 (0.2)	−0.19 (0.2)	−0.15 (0.3)

Table S3b. Module-trait relationships (T1). In each row, module of colors with different gene co-expression. In each column, clinical variables. The relationship between the two is shown and the statistical significance (p) in brackets.

geneSymbol	GS.Responder	p.GS.Responder	MM.turquoise	p.MM.turquoise
EBI3	-0.6433	0.0097	0.8027	0.0003
PDCD1LG2	-0.6460	0.0093	0.8845	0.0000
IL13RA2	0.6547	0.0081	-0.7070	0.0032
MERTK	0.6686	0.0064	-0.7204	0.0025
MICB	-0.7429	0.0015	0.7118	0.0029
CD70	0.6340	0.0111	-0.8365	0.0001
F2RL1	-0.7285	0.0021	0.7556	0.0011
HRAS	-0.7263	0.0022	0.7959	0.0003
CXCR5	-0.7177	0.0026	0.8482	0.0000
SMAD2	-0.7177	0.0026	0.8482	0.0000
NFATC1	-0.6036	0.0172	0.8990	0.0000
NOD1	-0.6033	0.0173	0.9238	0.0000
SPACA3	-0.6892	0.0045	0.9183	0.0000

Table S4a- Turquoise module- Information on genes associated with responder at T0.

GS.Responder: Gene Significance: correlation between gen expression and clinical variable (responder).
p.GS.Responder: p-value of GS.
MM: Module membership: degree of belonging of the gene to each module, measured by the correlation between the expression profile of the gene and the expression profiles of the rest of the genes in the module.

geneSymbol	GS.PFS	p.GS.PFS	MM.green	p.MM.green
PLAU	-0.5400	0.0308	0.8079	0.0002
SPA17	-0.5182	0.0397	0.9296	0.0000
TNFRSF10B	-0.6417	0.0074	0.8638	0.0000
GPI	-0.6079	0.0125	0.9135	0.0000
CCL24	-0.5796	0.0186	0.7532	0.0008
IFI16	-0.6354	0.0082	0.9293	0.0000
CCL8	-0.6252	0.0096	0.8367	0.0000
C4BPA	-0.5116	0.0428	0.8671	0.0000
CD209	-0.5867	0.0169	0.9345	0.0000
CFP	0.5059	0.0456	-0.7357	0.0012
CFI	-0.5665	0.0221	0.8711	0.0000
CD37	-0.7003	0.0025	0.8851	0.0000
TLR1	-0.6797	0.0038	0.7907	0.0003
NA	-0.6700	0.0045	0.9154	0.0000
TICAM2	-0.6623	0.0052	0.9717	0.0000

IL21	-0.6998	0.0025	0.9261	0.0000
CASP1	0.5455	0.0288	-0.8893	0.0000
CEBPB	-0.6357	0.0081	0.8469	0.0000
CXCL3	0.5708	0.0209	-0.8275	0.0000
LTBR	-0.5655	0.0224	0.7998	0.0002
CD1A	-0.6831	0.0035	0.7924	0.0003
EDC3	-0.6962	0.0027	0.9333	0.0000
BIRC5	0.6710	0.0044	-0.7791	0.0004
EOMES	-0.5281	0.0355	0.9427	0.0000
NA	0.6593	0.0055	-0.7138	0.0019
C7	-0.6197	0.0105	0.9131	0.0000
PAX5	-0.5133	0.0420	0.7229	0.0016
CXCL1	-0.5513	0.0268	0.9031	0.0000
ATG12	-0.6377	0.0079	0.8737	0.0000
TLR6	-0.5869	0.0169	0.8273	0.0000
IFNG	-0.6950	0.0028	0.8292	0.0000
IL21R	-0.5049	0.0461	0.8035	0.0002
NEFL	-0.5926	0.0156	0.8271	0.0000
ICAM1	-0.5885	0.0165	0.7477	0.0009
MAGEC2	-0.6062	0.0128	0.7843	0.0003
IL1RL1	-0.5449	0.0291	0.7576	0.0007
CSF3R	0.5410	0.0304	-0.7695	0.0005
IFIT2	0.6222	0.0101	-0.8191	0.0001
TAB1	0.6058	0.0129	-0.8305	0.0000
ULBP2	-0.5172	0.0402	0.7560	0.0007
HLA-DRB3	-0.6189	0.0106	0.7831	0.0003
CD46	-0.5139	0.0417	0.7353	0.0012
NA	0.6047	0.0131	-0.7797	0.0004
ATF2	-0.5458	0.0287	0.7282	0.0014

Table S4b- Green module- Information on genes associated with PFS at T1

geneSymbol	GS.LDH	p.GS.LDH	MM.brown	p.MM.brown
TANK	0.7748	0.0004	-0.9069	0.0000
CCL3L1	0.7725	0.0005	-0.8549	0.0000

HLA-DPA1	0.7442	0.0009	-0.9178	0.0000
CD1D	0.7389	0.0011	-0.9223	0.0000
CD3G	0.7175	0.0018	-0.9603	0.0000
PLAU	0.7152	0.0018	-0.8931	0.0000
CD48	0.7094	0.0021	-0.7553	0.0007
IFNB1	0.7004	0.0025	-0.8615	0.0000
DDX58	0.6890	0.0032	-0.8970	0.0000
BAGE	0.6790	0.0038	-0.8045	0.0002
CD14	0.6749	0.0041	-0.8626	0.0000
PSMB7	0.6729	0.0043	-0.9158	0.0000
CTSG	-0.6694	0.0046	0.9058	0.0000
TNFRSF10B	0.6588	0.0055	-0.8603	0.0000
PSMD7	0.6578	0.0056	-0.7728	0.0004
CYFIP2	-0.6575	0.0056	0.9295	0.0000
TIRAP	-0.6575	0.0056	0.9547	0.0000
BLNK	0.6572	0.0057	-0.9314	0.0000
IRAK4	0.6568	0.0057	-0.8075	0.0002
SPO11	-0.6564	0.0058	0.7994	0.0002
CARD9	-0.6476	0.0067	0.9356	0.0000
TLR3	-0.6454	0.0069	0.8714	0.0000
IL10RA	0.6431	0.0072	-0.8268	0.0000
CD55	0.6397	0.0076	-0.7589	0.0007
IL22	0.6372	0.0079	-0.8366	0.0000
HLA-A	0.6360	0.0081	-0.8100	0.0001
APOE	-0.6337	0.0084	0.9476	0.0000
CD33	-0.6205	0.0103	0.9213	0.0000
CCL27	-0.6192	0.0105	0.9226	0.0000
CSF2	0.6190	0.0106	-0.7693	0.0005
GPI	0.6142	0.0114	-0.7924	0.0003
CCL28	-0.6110	0.0119	0.7807	0.0004
DPP4	-0.6098	0.0121	0.9373	0.0000
CCR2	0.6090	0.0123	-0.7835	0.0003
ATG10	0.6090	0.0123	-0.9081	0.0000
CCL24	0.6084	0.0124	-0.8735	0.0000

TNFRSF11A	-0.6075	0.0126	0.8155	0.0001
CTCFL	0.6074	0.0126	-0.7538	0.0007
TNFSF18	-0.6014	0.0137	0.7803	0.0004
IFI16	0.6004	0.0139	-0.7305	0.0013

Table S4c- Brown module- Information on genes associated with LDH stratification at T1
